# Recent advances in JAK inhibitors for the treatment of metabolic syndrome

**DOI:** 10.3389/fphar.2023.1245535

**Published:** 2023-08-24

**Authors:** Debora Collotta, Maria Paola Franchina, Virginia Carlucci, Massimo Collino

**Affiliations:** ^1^ Department of Neuroscience “Rita Levi-Montalcini”, University of Turin, Turin, Italy; ^2^ Chemsafe S.r.l., Turin, Italy

**Keywords:** JAK-STAT pathway, JAKi, metabolic syndrome, inflammation pharmacology, inflammation-releted diseases

## Abstract

With an epidemic spread, metabolic syndrome represents an increasingly emerging risk for the population globally, and is currently recognized as a pathological entity. It is represented by a cluster of different conditions including increased blood pressure, high blood sugar, excess body fat around the waist and abnormal cholesterol or triglyceride levels. These conditions lead directly to several disorders, including obesity, dyslipidemia, hyperglycaemia, insulin resistance, impaired glucose tolerance and hypertension causing an increase in cardiovascular risk and in particular atherosclerotic disease. Despite efforts to promote healthier lifestyles through exercise, reduced caloric intake, and improved dietary choices, the incidence and prevalence of metabolic syndrome continue to rise worldwide. Recent research has highlighted the involvement of signaling pathways in chronic inflammatory conditions like obesity and type 2 diabetes mellitus, revealing the significance of the JAK/STAT pathway in atherosclerotic events. This pathway serves as a rapid membrane-to-nucleus signaling module that regulates the expression of critical mediators. Consequently, JAK inhibitors (JAKi) have emerged as potential therapeutic options for metabolic diseases, offering a promising avenue for intervention. The aim of this review is to shed light on the emerging indications of JAK inhibitors in metabolic syndrome, emphasizing their potential role in attenuating associated inflammatory processes, improving insulin sensitivity, and addressing cross-talk with the insulin pathway, with the intention of contributing to efforts in the field of inflammation pharmacology.

## 1 The JAK-STAT signaling pathway: an overview

The Janus kinase-signal transducer and activator of transcription (JAK-STAT) pathway is crucial for many vital cellular processes, including the expression of inflammatory mediators. Indeed, the role of JAK-STAT is to transmit, through interaction with transmembrane receptors, information received from extracellular space signals, thereby mediating a downstream message to the nuclear compartment. Specifically, the receptors involved in this mechanism are termed type I and type II and are capable of binding cytokines like interleukins (IL-6, IL-10, IL-12), interferons and hormones (Krueger et al., 2022).

In mammals, there are four recognized members of the JAK family: JAK1, JAK2, JAK3, and TYK2. Each has different receptor selectivity and consists of an N-terminal domain responsible for protein-protein interaction, a Src-homology 2 (SH2) domain whose role is the activation and dimerization of STATs. In addition, a central pseudo-kinase domain having a regulatory role and a protein tyrosine kinase (PTK) domain in the C-terminal region is responsible for the phosphorylation of a specific tyrosine residue. The pathway is activated when cytokines bind to receptors, leading to Jak activation through phosphorylation. Activated JAK, in a cascade, causes transphosphorylation of specific tyrosine residues, generating a docking site for STAT. In the absence of specific receptor stimulation, STAT proteins are in fact inactive at the cytoplasmic level as transcription factors. Instead, they are rapidly activated following the response of the receptor-ligand coupling and are recruited by JAK, at the SH2 domain and phosphorylated (Seif et al., 2017). Phosphorylated STAT leaves the docking site forming homodimers and heterodimers, which are able to translocate towards the nucleus and bind specific DNA sequences, defined as DNA-binding elements (Hu et al., 2021). The result is the activation or suppression of the transcription of target genes, an intrinsic property of STAT dimers which are capable of recruiting nuclear coactivators able to allow chromatin modifications and communication with the core promoters (Aaronson and Horvath, 2002). The STAT family consists of 7 members: STAT1, STAT2, STAT3, STAT4, STAT5a, STAT5b, and STAT6. As for JAK, an N-terminal region determining the dimerization is defined also for STAT. Moreover, it has been described an SH2 domain responsible for cooperation with JAK, protein-protein interaction and subsequent nuclear translocation, as well as the DNA-binding domain (DBD) capable of recognizing and binding specific DNA sequences corresponding to the regulation of target genes and the transcriptional activation domain (TAD). Finally, the C-terminal domain, fundamental in the activation of STAT, presents a highly conserved region of phosphorylated tyrosines and serines (Xin et al., 2020).

It has to be acknowledged that the complex process of JAK-STAT activation and signaling can be regulated through various processes such as dephosphorylation capable of attenuating the activity of STAT, or through receptor internalization mechanisms in endocytic vesicles with consequent degradation and turn off the pathway. JAK also has its own inhibitors, crucial in the negative feedback mechanism, which go by the name of cytokine signal suppressor proteins (SOCS) in charge of inactivating kinases. In the same way, inhibitory proteins of activated STAT (PIAS) are capable of recognizing and binding STAT dimers while avoiding binding with DNA sequences (Aaronson and Horvath, 2002).

The tissue distribution of JAK isoforms was analyzed using the Human Protein Atlas (HPA), which is an open access resource for the exploration of specific human genes and proteins. The JAK1, JAK2, and JAK3 proteins have been shown to have a rather heterogeneous tissue expression, including liver, adipose tissue and skeletal muscle, while Tyk2 is mainly expressed in immune cells (Proteinatlas, 2023). Moreover, recent studies have highlighted the importance of JAK-STAT signaling in the regulation of energy metabolism in the brain. JAK2 and STAT3 are highly expressed in hypothalamic neurons, and their activation has been shown to regulate food intake, energy expenditure, and glucose homeostasis (Koch et al., 2008). Pivotal role, in the link between JAK/STAT and feeding is played by leptin, a hormone synthesized by adipocytes and involved in the regulation of caloric expenditure and appetite at the hypothalamic level. Indeed, JAK2 undergoes a process of autophosphorylation as a result of binding of the hormone to the b-isoform of its receptor. In turn, phosphorylation of JAK2, as previously illustrated, involves phosphorylation of downstream molecules including STAT3 (Gurzov et al., 2016). A dense communication network is thus activated between leptin, JAK2 and STAT3, which cascade to regulate the expression of the reverse neuropeptides: proopiomelanocortin (POMC) and agouti-related protein (AgRP) (Zhang et al., 2008). Indeed, STAT3 activation increases the expression of POMC in the reduction of appetite and food consumption, while it reduces AgRP and NPY (neuropeptide Y) with anorectic functions. I addition, also in this pathway, phosphorylation of STAT3 induces constitutive expression of the suppressor SOCS3, which in fact exerts negative feedback on the entire leptin pathway (Gurzov et al., 2016). It thus appears well known that JAK-STAT is required in normal food intake homeostasis, reducing appetite and increasing energy expenditure. At the same time, however, it is known how dysregulation of this pathway can result in metabolic conditions and obesity; just think of the hyperactivation of JAK-STAT3-SOCS in the setting of leptin resistance (Wunderlich et al., 2013; Qurania et al., 2018). Although several studies show that knockout mice for the leptin gene or for STAT3, develop obesity, precisely because of altered activation of the previously described pathway, modulation of JAK-STAT finds itself at the center of a conflicting condition for another property that has emerged: the promotion of UCP-1 and the consequent induction of the browning phenomenon. Indeed, the latter evidence, obtained mainly *in vitro*, has drawn attention to the possibility of further study of JAKi in metabolic contexts, being strongly correlated with the development of pathologies directly affecting metabolic organs (Dodington et al., 2018; Qurania et al., 2018). Given the increased attention paid to this pathway over the last few years, the dysregulation of which has seen it implicated in conditions of metabolic alteration such as obesity and type 2 diabetes, there have been increasing studies to target the various subjects involved and develop new therapeutic tools (Dodington et al., 2018). Initially, monoclonal antibodies binding specific cytokines or their receptors were tested (thus acting upstream of the signaling pathway), but difficulties at the clinical level have been encountered. Indeed, by indiscriminately blocking the upstream pathway, a range of adverse effects emerged due to the numerous cytokines involved or, at times, a lack of response from the patients (Gadina, 2013; Schwartz et al., 2016; Schwartz et al., 2017).

Recent developments in selective JAK inhibitors have revolutionized research, due to the ability of attenuating the unfavorable conditions of patients, acting on a less broad spectrum of cytokines and consequently reducing side effects (Kim, 2020). To date, the pharmacological manipulation of the JAK-STAT pathway by inhibitors appears to be a promising therapeutic approach for the treatment of metaflammation: a condition of chronic, low-grade systemic inflammation. It is mostly driven by a range of dietary factors (such as saturated fatty acids and sugars), and consequently, characteristic of patients with obesity, type 2 diabetes, nonalcoholic fatty liver disease (NAFLD), increasing cardiovascular risk (Charles-Messance et al., 2020). Actually, it has already been approved for rheumatoid arthritis, ulcerative colitis and psoriasis (Schwartz et al., 2017).

## 2 JAK-STAT inhibitors and their clinical use

The targeting of the JAK/STAT family has proven effective in various clinical conditions, revolutionizing the management of e immune and inflammatory diseases. These small inhibitory molecules also called JAKi (Janus Kinase Inhibitors) or Jakinibs have been recognized and investigated since the 90 s. In less than 20 years, 9 molecules have been approved by the FDA and others undergoing clinical trials for various indications (Winthrop, 2017) (Table 1). JAK inhibitors can be divided into two generations based on their selectivity. The first generation JAKi, lack specificity towards a particular isoform (e.g., Tofacitinib and Baricitinib), while the second generation JAKi, exhibit high selectivity (e.g., Filgotinib and Upadacitinib) (Shawky et al., 2022).

**TABLE 1:** State of the art of approved JAKi and their therapeutic indications.

Drug	Selectivity	FDA/EU approved indications	Indications under phase II/III clinical trials	Investigation studies in cardiometabolic disease
Ruxolitinib	JAK1, JAK2	Myelofibrosis; Acute graft-versus-host disease; Polycythaemia vera	Alopecia areata; Vitiligo; Essential thrombocythemia; Coronavirus disease 2019; Atopic dermatitis	Type 1 diabetes
Tofacitinib	JAK1, JAK3	Rheumatoid arthritis; Ulcerative colitis; Polyarticular juvenile idiopathic arthritis	Crohn’s disease; Alopecia areata; Dermatomyositis; Atopic dermatitis; Coronavirus disease 2019; Ankylosing spondylitis	Obesity, insulin resistance and type 2 diabetes
Baricitinib	JAK1, JAK2	Rheumatoid arthritis	Lupus; Atopic dermatitis; Coronavirus disease 2019; Alopecia areata	Diabetic kidney disease and insulin resistance
Peficitinib	JAK3	RA (Japan) arthritis.	Rheumatoid arthritis	
Fedratinib	JAK2	Myelofibrosis	AML	
Upadacitinib	JAK1	Rheumatoid arthritis	Crohn’s disease; Ulcerative colitis; Aatopic dermatitis; active psoriatic arthritis; Nkylosing spondylitis	
Filgotinib	JAK1	RA (EU approved)	Inflammatory bowel disease; A ctive psoriatic arthritis; Ankylosing spondylitis	
Delgocitinib	JAK1, JAK2, JAK3, TYK2	Atopic dermatitis	Alopecia areata; Chronic hand eczema; Lupus erythematosus	
Gandotinib	JAK2		Myeloproliferative neoplasms; Polycythemia vera; Essential Thrombocythemia	
Lestaurtinib	JAK2		Acute myeloid leukemia	
Momelotinib	JAK1, JAK2		Myelofibrosis	
Pacritinib	JAK2	Myelofibrosis in adult patients with thrombocytopenia		
			Myelofibrosis; Acute myeloid leukemia	
Deucravacitinib	TYK2	Psoriasis	Psoriatic arthritis; Inflammatory bowel disease; systemic lupus erythematosus	
Abrocitinib	JAK1	Refractory, moderate-to-severe atopic dermatitis	Atopic dermatitis	
Itacitinib	JAK1		Arthritis; Inflammatory bowel disease; Aacute graft-versus-host disease; CRS during CAR-T therapy; B-cell lymphoma	
Decernotinib	JAK3		Rheumatoid arthritis	
Gusacitinib	JAK1, JAK2, JAK3, TYK2		Atopic dermatitis	
Cerdalatinib	JAK1, JAK2, TYK2		Non-Hodgkin lymphoma	

Specifically, several pathologies have been associated with the increased activity of different isoforms of the Janus kinase family, and among these JAK1 seems to be widely involved. Being associated with IL-6, IL-2, IL-4, IL-15, IFNα, and IFNγ binding receptors, JAK1is implicated in inflammatory conditions, innate immunity responses, lymphocyte activity and antiviral activity (Traves et al., 2021). Its inhibition (often associated with JAK2 inhibition) has so far been shown to be effective in the treatment of rheumatoid arthritis and itch-associated psoriatic arthritis (Shawky et al., 2022). The JAK2 isoform shares largely the same implications as JAK1 and has additionally been associated with erythropoiesis and thrombopoiesis. JAK3 is instead exclusively associated with the common receptor chain γ (γc) cytokine family, which includes interleukin-2 (IL-2), IL-4, IL-7, IL-9, IL-15, and IL-21 (Suzuki et al., 2000; Lin and Leonard, 2018), while TYK2 coupled to JAK1 is involved in the innate immune response (Traves et al., 2021).

Recent evidence of the clinical significance of Jak inhibitors was demonstrated by Tofacitinib and Baricitinib’s effective blockade of cytokine storm in COVID-19, leading to improved outcomes among hospitalized patients (Khaledi et al., 2022; COVID-19 treatment guidelines, 2023).

## 3 Role of JAK-STAT pathway in metabolic diseases

Considering the close link between the immune and inflammatory response, recent efforts have been made to explore the potential of JAKi in managing metabolic syndrome.

In recent years, the significance of an epidemic emergency and its diverse manifestations has prompted investigation into molecular pathways that can adress and mitigate the risk to the cardiovascular system. The latter involves non-modifiable factors such as age and sex, but mostly modifiable biological risk factors and consequently preventable factors including fasting blood glucose, insulin resistance, atherogenic blood lipid profile and elevated blood pressure (Vanuzzo et al., 2008). The studies conducted on the JAK-STAT pathway have allowed it to be positioned among the potential therapeutic targets for the treatment of obesity and diabetes. JAK/STAT signaling pathway plays a critical role in the regulation of target organs of metabolism such as liver, muscles, fat and pancreas (Dodington et al., 2018). Recent targeted researches have brought to light numerous interactions between the JAK-STAT pathway and other mediators, including e the insulin cascade b and leptin, a hormone regulating caloric expenditure and appetite. Specifically, a population study was able to demonstrate how the JAK2 polymorphism was associated with the increase in adipose tissue and in waist circumference, as a result of the leptin-JAK2-STAT3 interaction (Ge et al., 2008). While in the article by Shi et al. (2012), the authors indicate that JAK2 deletion in the liver protects mice against whole-body insulin resistance and glucose intolerance, it concurrently promotes hepatic steatosis but, surprisingly, did not evolve into steatohepatitis.

One possible explanation for these paradoxical findings could be the interaction and crosstalk between the signaling pathways involved. For instance, the authors suggest that the attenuated insulin signaling in the liver might stimulate compensatory β-cell proliferation in response to high-fat diet feeding, contributing to the resistance against the development of glucose intolerance.

On the other hand, in a separate study, Shi et al. (2014) demonstrated that adipocyte-specific JAK2-KO mice were susceptible to high-fat diet-induced obesity and diabetes. Lipid accumulation in adipose tissue was initially benign with no adverse effects on metabolic variables. However, the progressive deposition of lipids eventually exceeded the storage capacity of existing adipocytes, leading to the release of NEFA into the circulation and the ensuing metabolic consequences as mice grew older. These results suggest that JAK2 may play different roles in different tissues and metabolic conditions. However, further research is needed to fully clarify the underlying mechanism and the potential crosstalk between the different signaling pathways involved in the context of various metabolic conditions.

In conclusion, the intricate role of JAK2 in the different signaling pathways involved in liver metabolism, adipose tissues, and pancreatic β-cells suggests that crosstalk mechanisms and interactions between these pathways are essential to fully understand the effects of JAK2 in metabolic regulation. Further studies will be necessary to delineate these mechanisms more comprehensively and their role in metabolic processes.

Inflammatory conditions that persist over time, such as in diabetes and obesity, directly contribute to the development of atherosclerosis and other cardiovascular diseases. The implication of JAK-STAT appears clear in the context of metabolic conditions as previously described, while its role on the cardiovascular system remains partly unexplored and more difficult to understand. A cytokine that plays a pivotal role in the development of atherosclerosis is again IL-6 which, by binding its receptor, initiates a series of cellular events through the phosphorylation of JAKs and the activation of STATs (especially STAT3). The latter, through nuclear translocation, activates the angiotensinogen promoter gene in cardiomyocytes which in turn leads to the production of angiotensin II capable of stimulating the production of cardiotrophin-1, a member of the IL-6 family responsible for the development of cardiac hypertrophy. At the same time, IL-6, through JAK/STAT, appeared able to promote endothelial activation with increased permeability towards leukocytes and adhesion molecules, increasing thrombotic risk (Baldini et al., 2021). As the injury progresses the complex network of molecules and mediators involved increases, further worsening the inflammatory condition. Among them, activated macrophages are capable of producing reactive oxygen species (ROS) that increase the oxidation of low-density lipoproteins (LDL), a phenomenon directly related to the accumulation of cholesterol crystals (Baldini et al., 2021).

## 4 Cross-talk between JAK-STAT and insulin pathways

**FIGURE 1:**
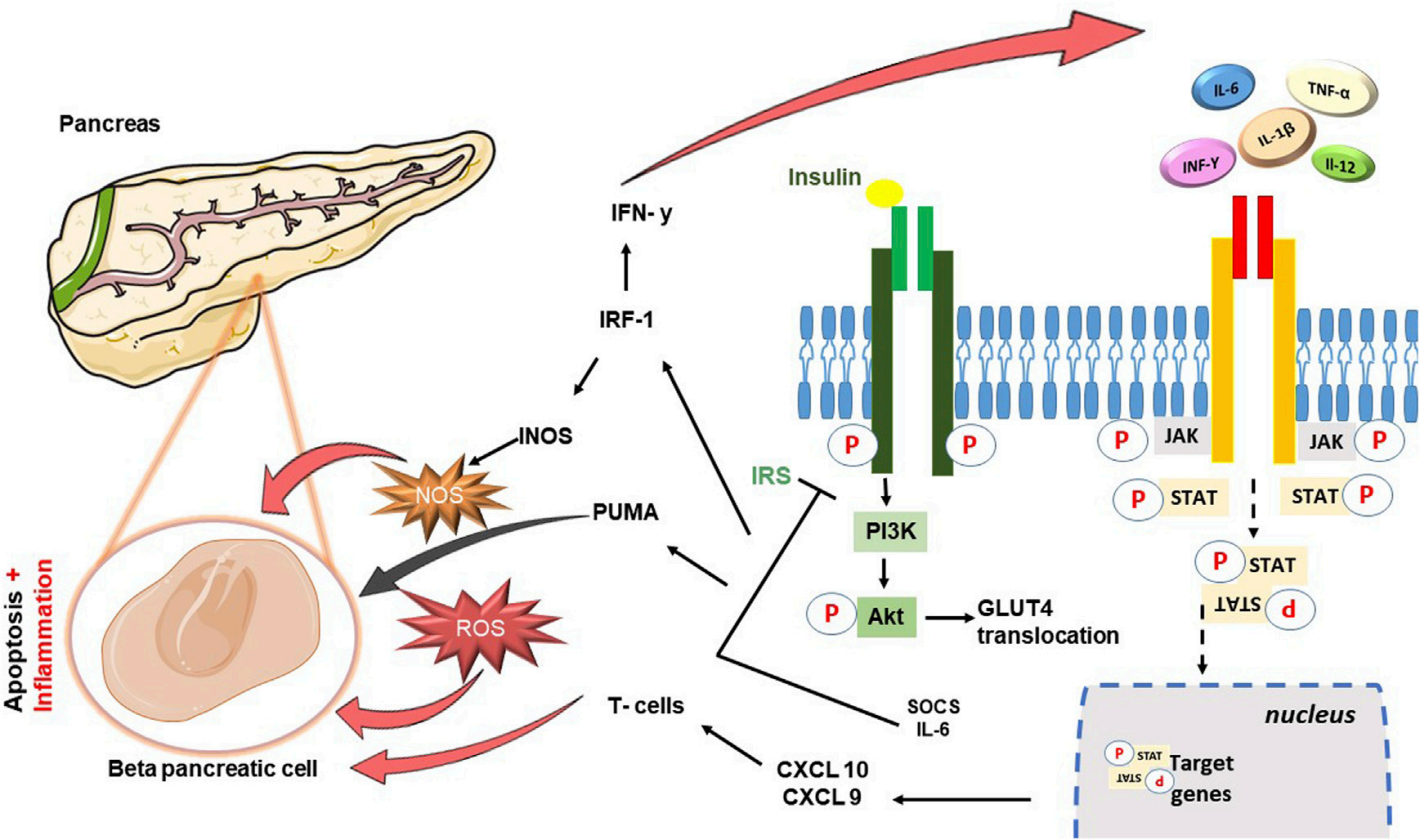
Schematic diagram showing cross-talk between JAK-STAT cascade and Insulin signaling pathway. In brief insulin hormone binds its receptor with tyrosine kinase activity: the propagation of the signal involves the binding between the insulin receptor substrate (IRS) and the phosphoinositide-3-kinase (PI3K), leading to the activation of Akt, and consequently the translocation of GLUT4. JAK-STAT pathway can associate with the insulin receptor, be phosphorylated in insulin-sensitive tissues and its hyperactivation contribute to the reduction of phosphorylation of Akt. IL-6 leads to an increase in the levels of cytokine signaling inhibitor proteins SOCS1 and SOCS3, resulting in impairment of the insulin signaling pathway through binding to IRS-1 and IRS-2. The damage is perpetuated precisely to the detriment of the β-pancreatic cells responsible for the production of insulin: β-cell apoptosis follows the increase in the expression of apoptotic factors including p53 upregulated modulator of apoptosis (PUMA), directly dependent on JAK2-STAT1 activation. Moreover, activity of STAT1 is responsible for the transcription of target genes such as interferon regulatory transcription factory (IRF)-1, inducible nitric oxide synthase (iNOS) and other chemokines (CXCL9, CXCL10) important in attraction of T cells and their direction to the pancreas.

Within the metabolic context explored in this review, a significant finding is the cross-talk between the JAK-STAT pathway and the insulin cascade, which emerged from different studies. Insulin signaling is mediated by a highly integrated network of processes that regulate very complex mechanisms. Normally, insulin binds its receptor IR (Insulin Receptor) with tyrosine kinase activity. This interaction triggers a series of sequential events designed to stimulate and release insulin itself (Taniguchi et al., 2006). The propagation of the signal subsequently involves the binding between the insulin receptor substrate (IRS) and the phosphoinositide-3-kinase (PI3K), leading to the formation of phosphatidylinositol-(3,4,5)-triphosphate (PIP3) and activating thereafter Akt. Akt, following phosphorylation, translocates towards the cytosol, promoting the storage of glucose in the form of glycogen and the translocation of GLUT4 (Lizcano and Alessi, 2002).Studies conducted in the metabolic field have demonstrated how protracted high-fat diets (High Fat Diet–HFD) and therefore chronic inflammatory conditions, lead to glucose intolerance and systemic insulin resistance in liver and muscle tissues IRS-1 (Lee et al., 2011) is among the most involved substrates, which can undergo phosphorylation of specific amino acid sites with consequent changes in the state of activation by inflammatory cytokines TNF-α and free fatty acids (FFA) (Schmitz-Peiffer and Whitehead., 2003). Among these, the JAK-STAT pathway also plays a role in the alteration of the insulin pathway (Figure 1): it can associate with the insulin receptor, be phosphorylated in insulin-sensitive tissues and its hyperactivation contribute to the reduction of phosphorylation of Akt (Thirone et al., 2006). More specifically, JAK2 appears to be particularly involved with further mechanisms, in addition to the most just described, which involve both IRS-1 and IRS-2 (Ge et al., 2008). IL-6 plays a particularly significant role in conditions like obesity, leading to elevated levels of cytokine signaling inhibitor proteins SOCS1 and SOCS3, which are primarily found in insulin-sensitive peripheral tissues like white adipose tissue, liver, and muscle, At the molecular level, the increase in SOCS1 and SOCS3 results in impairment of the insulin signaling pathway through binding to IRS-1 and IRS-2 (Shi et al., 2016). This phenomenon leads to the ubiquitination of IRS-1 and IRS-2 and to their degradation and the inevitable consequent onset of diabetes (Senn et al., 2003; Howard and Flier, 2006). However, a clarification needs to be made to avoid confusion between the role of SOCS suppressors and JAKi: while SOCS proteins can be likened to JAK inhibitors due to their ability to inhibit JAK/STAT signaling, it is crucial to acknowledge the complexity of this relationship and the different roles played by specific SOCS members. For instance, SOCS-1 and SOCS-3 may inhibit insulin activity and contribute to insulin resistance, while SOCS-6 and SOCS-7 seem to have a protective role against diabetes (Suchy et al., 2013). The distinct roles of different SOCS proteins in metabolic regulation are a subject of ongoing research, and further studies are needed to fully understand their individual contributions to insulin resistance, glucose metabolism, and the development of diabetes. As our understanding of these mechanisms advances, it may offer new insights into potential therapeutic targets for managing metabolic disorders and diabetes. JAK/STAT signaling can lead to insulin resistance when overactivated, and is also involved in inflammatory pathways associated with metabolic syndrome. I nhibiting JAK/STAT signaling using JAK inhibitors may reduce inflammation and pro-inflammatory cytokines production, known contributors to insulin resistance and metabolic dysfunction. Furthermore, recent studies using tissue-specific knockout mice have shed light on the role of JAK/STAT signaling, particularly the JAK2 pathway, in peripheral metabolic organs such as adipose tissue, liver, skeletal muscle, and pancreas. The loss of JAK2 in specific tissues has been associated with resistance to diet-induced metabolic stress and protection against insulin resistance and inflammation (Shi et al., 2012; Desai et al., 2017; Corbit et al., 2017). In contrast, loss of JAK3 in mice results in significant body weight increase, associated with impaired glycemic homeostasis, hyperinsulinemia and early symptoms of liver steatosis (Mishra Jet al. 2015). These findings suggest that the effects of JAK inhibition on insulin signaling and metabolism may be context-dependent and influenced by tissue-specific JAK isoforms. To confirm the implication in diabetes, this series of events and obstacles in insulin signaling also prevents the translocation of GLUT-4 from the vesicles to the cell surface hindering glucose uptake. Moreover, the overexpression of SOCSs proteins and high cytokines levels stimulate the production of acute phase hepatic proteins, including serum amyloid A (SAA), which further confirms the presence of inflammation (Bako et al., 2019). This inflammatory environment damages t β-pancreatic cells, responsible for insulin production, exacerbating the development of diabetes. Studies conducted by Moore et al. (2011) and Stanley WJ et al. (Stanley et al., 2015) demonstrated how human and mouse models underwent β-cell apoptosis following the increase in the expression of several apoptotic factors including p53 upregulated modulator of apoptosis (PUMA), directly dependent on JAK2-STAT1 activation. Same studies were then able to demonstrate further damaging activity of STAT1 in the transcription of target genes such as interferon regulatory transcription factory (IRF)-1, inducible nitric oxide synthase (iNOS) and other chemokines (CXCL9, CXCL10) important in attraction of T cells and their direction to the pancreas. In conclusion, the inflammatory environment promotes the formation of ROS further exacerbating the production of IFN-γ and worsening the pancreatic damage, inevitably leading to a condition of extreme facility for the development of diabetes (Gurzov et al., 2016).

## 5 The role of JAKi in metabolic disorders

**FIGURE 2:**
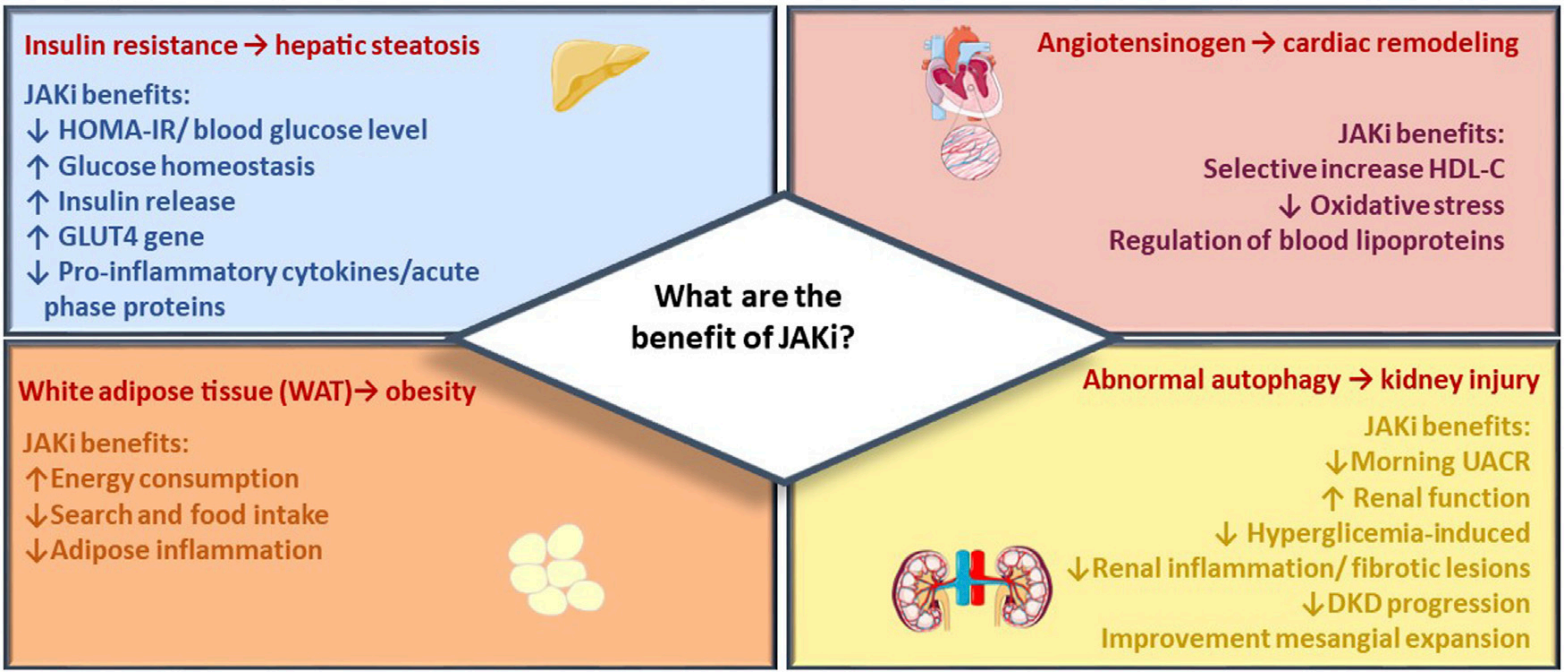
JAK inhibitor metabolic effects. The figure summarizes main beneficial effects of JAK inhibitors across target organs of metabolism, including reduction of CV events, renal protection, and improvement of metabolic control in diabetes and obesity.

In the present section, the most recent evidence emerging in several studies is discussed with the aim to strengthen the implication of the JAK/STAT pathway in metabolic dysregulations (Figure 2). The most recent studies are based on data collected so far on “older” molecules, which are involved in studies in the cardiometabolic field. Specifically, Tofacitinib, Baricitinib, and Ruxolitinib have been extensively studied in both pre-clinical and clinical trials due to their well-established safety profiles resulting from their prolonged and extensively tested usage.

### 5.1 Tofacitinib

Recent studies seem to highlight a potential role of JAKi in the browning process of adipose tissue and, consequently, in fighting obesity. Adipose tissue is involved, as previously described, in the regulation of the energy balance and in the homeostasis of each individual. Dodington DW and others demonstrated in an *in vitro* study the ability of Tofacitinib (JAK1/3 inhibitor) to induce the browning process through the suppression of IFN ɣ (Dodington et al., 2018). Subsequently, the study conducted by Qurania KR et al. confirmed the previously mentioned ability of Tofacitinib and Ruxolitinib (JAK1/2 inhibitor) *in vivo*, in a mouse model, by elucidating the underlying mechanism. Treatment with JAKi indeed showed an increase in the mRNA expression of UCPs and in particular UCP-1, an uncoupling protein of oxidative phosphorylation, involved in the browning process. In fact, it acts on the production of ATP by inducing the uncoupling of oxidative phosphorylation, with the result being the release of energy in the form of heat (Qurania et al., 2018). Although JAK inhibition promoted UCP1, treated mice showed increased food intake, while maintaining the same body weight and adiposity. However, JAKi-treated mice exhibited increased thermogenic capacity and reduced levels of serum triglycerides and free fatty acids (Qurania et al., 2018). In accordance with what has already emerged from the literature review so far, the JAK-STAT pathway proves to be as interesting as insidious in the mechanisms involved in treating diabetes and obesity among metabolic diseases. Indeed, another study has pointed out the necessity of JAK2 in BAT for the induction of UCP1 itself and thus in energy expenditure (Shi et al., 2016). This apparently countercurrent mechanism to the previously highlighted implication could be explained by recalling the important differences between BAT and WAT, the latter precisely involved in the browning process promoted by JAKi. Indeed, it is well known that BAT and WAT differ in cell morphology, gene expression, and metabolic functions (Miura, 2015). JAKi’s potential as a therapeutic strategy for obesity is worth considering in the clinic, particularly its role in browning Indeed, a study published in Nature cell biology hypothesized the possibility of directing JAKi or similar compounds selectively to white adipocytes, exploiting precisely the thermogenic capacity and preventing their systemic spread (Moisan et al., 2014) The potential of Tofacitinib has also been investigated in the field of insulin resistance and type 2 diabetes in both the pre-clinical and clinical settings. Scrupulous research activities have shown that an increased risk of insulin resistance (IR) frequently occurs in patients with rheumatoid arthritis (RA) as a chronic inflammatory condition associated with the dysregulated release of pathogenic cytokines. Indeed, the literature shows the involvement of TNF-α in the pathogenesis of IR. The mechanism of interference is related to the phosphorylation of the serine residue and the reduction of tyrosine phosphorylation of IRS-1 and the β subunit of the insulin receptor itself. IL-6 in the same way is able to reduce the degree of tyrosine phosphorylation of the same insulin receptor; while the involvement of IL-1β lies in the activation of the NF-kB inflammatory molecular pathway linked to the insulin pathway through serine phosphorylation. The known ability of JAK/STAT inhibitors to reduce numerous pro-inflammatory cytokines, has led Tofacitinib to be included in different studies aimed at the treatment of IR (Wang and Tsai, 2022). A first *in vivo* investigation was conducted by Bako HY et al. in a study published in 2019, where the team tested the efficacy of tofacitinib in combination with aspirin in contrasting the condition of chronic inflammation directly related to IR development. The rats, after undergoing type 2 diabetes induction by administration of 10% fructose, were treated with different doses of tofacitinib, aspirin and the two drugs in combination for 9 weeks. The analyzes of the results showed the ability of both treatments to significantly (*p* < 0.05) reduce the investigated pro-inflammatory cytokines and SAA. Even more promising data were obtained from the combination of the two drugs which showed a significant reduction of the above values, HOMA-IR and blood glucose levels and a significant improvement in glucose homeostasis, insulin release, HOMA-β and expression of the GLUT4 gene, demonstrating the important involvement of both the JAK/STAT pathway and NF-kB (Bako et al., 2019). The retrospective study conducted by Wang CR et al. carried out from April 2017 to March 2021 and published in 2022 on active RA patients, allowed us to confirm the data. Patients, after receiving tofacitinib treatment for 24 weeks, showed significant reductions in IR based on the assessment of HOMA indices. These findings are significant not only for type 2 diabetes which is closely connected to IR, but also to the pathophysiology of obesity, both being conditions characterized by high levels of circulating cytokines and consequently a latent level of low-grade inflammation (Wang and Tsai, 2022).

Further interest in JAKi, in particular Tofacitinib, in the inflammatory and cardiometabolic field is easily attributable to the link between this class of molecules and the lipid profile. Indeed, the inhibition of the JAK/STAT pathway is able to contribute to the release of lipids by modulating the expression of hepatic cell receptors and the synthesis of ATP-binding cassette transporters (ABCA1) (Kotyla et al., 2020). ABCA1 are specific transporters located on macrophages, responsible for the efflux of cholesterol, transferring it in the form of HDL. It is therefore easy to understand how the outflow operated by the macrophages is fundamental for the prevention of the formation of atherosclerotic plaques (Pérez-Baos et al., 2017). At the same time, tofacitinib was found, in another study, to be able to significantly reduce the levels of IFN-γ. This cytokine, through the JAK-STAT pathway, is able to regulates more than 2,300 genes, but of particular interest is its role, currently under study, in the modification of blood lipoproteins, in the promotion of the development of atherosclerotic lesions, in the induction of oxidative stress and therefore cardiovascular events (Kotyla et al., 2020). To confirm this, in a multiple phase III study lasting 24 months and two open-label long-term extensions (LTE) conducted by the Charles-Schoeman team, tofacitinib administered to patients with RA was associated to a decrease in the incidence of cardiovascular events (Charles-Schoeman et al., 2016). The same authors, through a *post hoc* analysis carried out after 7 years on the same studies, affirmed that the drug was able to selectively increase the levels of HDL-C, further confirming the beneficial potential of the drug (Charles-Schoeman et al., 2019).

### 5.2 Baricitinib

Baricitinib, like tofacitinib, has been studied in the cardiometabolic field to explore its potential. In a clinical study on patients with Diabetic kidney disease (DKD), researchers investigated the role of JAK1 and JAK2 in DKD onset and progression. The study revealed that an increase in the expression and activity of these isoforms was involved in both onset and progression of DKD. Moreover, significant studies were presented suggesting an interaction between the JAK/STAT pathway and angiotensin signaling, with particular interest in evidence of JAK2 activation (Navarro-González et al., 2011). Based on this evidence, a first phase 2 randomized clinical trial was conducted to examine the effects of a JAKi on DKD pathology. The study, which featured 24 weeks of treatment with the inhibitor Baricitinib in participants at high risk for disease progression, met its primary endpoint by demonstrating significant reductions in morning urine albumin-creatinine ratio (UACR) across the entire duration of the experiment. Therefore, JAK1/JAK2 inhibition is shown to be a potential tool for improving health outcomes in patients inhibiting hyperglycemia-induced damage to kidney cells, improving renal function, reducing renal inflammation and fibrotic lesions and slowing the progression of DKD (Chen et al., 2021). On these bases, we have recently published the results obtained from a complex study focused on the analysis of baricitinib as a pharmacological tool in a model of diet-induced metaflammation. The study demonstrated the ability of the JAK inhibitor to correct abnormalities in insulin signaling in the liver and skeletal muscle, shedding further light on the pilot role of JAK2 in the regulation of different metabolic processes implicated in the development of diabetes and obesity. When compared to mice exposed to a high-fat, high-sugar diet, mice treated with baricitinib indeed showed a reduction in plasma insulin and leptin levels and a restoration of glucose-dependent insulinotropic polypeptide (GIP) and ghrelin levels (Collotta et al., 2020). The former is an important hormone with orexigenic properties, responsible for the search and intake of food and the decrease in energy consumption, as well as the protagonist in the modulation of lipid storage in adipose tissue (Thondam et al., 2020). Similarly, ghrelin plays a key role in metabolic processes, and its deficiency has been associated with worsened diet-induced obesity, insulin resistance and adipose inflammation (Colldén et al., 2017). These results were consequently demonstrated, by an improvement in myosteatosis (with reduction in triglyceride levels, total cholesterol and LDL/HDL ratios), mesangial expansion and associated proteinuria; confirming protection at the muscle and kidney level as well as a reduction of the overall inflammatory picture (Collotta et al., 2020). As documented in patients with rare molecular defects in the insulin signaling pathway (Semple et al., 2009), metabolic dyslipidemia is the result of selective post-receptor hepatic insulin resistance. Thus, we speculate that the preservation of insulin sensitivity may also account for improved systemic lipid profiles. The improved insulin sensitivity could potentially lead to better regulation of lipid metabolism and contribute to the observed changes in the lipid profile.

## 6 JAK/STAT follow up at cardiovascular level

The Canakinumab Anti-Inflammatory Thrombosis Outcomes Study (CANTOS) has revealed interesting perspectives on the use of JAK inhibitors. The study demonstrates the potential to reduce cardiovascular disease risk by targeting interleukin-1β with monoclonal antibodies. Multiple inflammatory mediators, particularly cytokines such as IL-6, IL-1β, TNF-α, and IFNγ. play important roles in atherosclerosis and need to be monitored and targeted. This paves the way for JAK as a potential therapeutic target in atherosclerosis treatment (Lembo, 2018).

It is also interesting what was discovered by other multiple studies: a beneficial and protective role of STAT3 in the heart. The study, specifically conducted on the heart, saw the use of STAT3 cardiac myocyte knockout mice (STAT3-KO). STAT3-KO mice subjected to 1-h ischemia followed by 24-h reperfusion experienced significantly increased infarct size. In wild type (WT) mice, however, pre- and post-ischemic conditioning protocols resulted in a significant reduction in myocardial infarction size and an improvement in cardiac function, demonstrating the protective and pro-survival effects of STAT3 (Frias and Montessuit, 2013). In the same study, however, a contrasting role of STAT1 was reported, which unlike what has just been defined for STAT3, was associated with deleterious effects. In the studies by McCormick et al., STAT1 shutdown using a knockout mouse model has been shown to have post-ischemia reperfusion benefits with reduction in myocardial infarction size and reduction in autophagy (McCormick et al., 2012). Moreover, the study conducted by Hui-Hui Guo et al., observed a lack of involvement of STAT5 in cardioprotection following ischemia-reperfusion. The team was also able to explain why the particular action of STAT3 appears to diverge from the other isoforms at this point. In fact, the link between STAT3 and matrix metallopeptidase 3 (MMP-3) has been recognized, which in turn codes for the extracellular matrix degradation enzyme (MMP-3), responsible for tissue remodeling, wound repair and with the progression of atherosclerotic phenomena (Guo et al., 2021). Recent observations on patients with COVID-19 revealed interesting findings. The combination of Baricitinib 4 mg/day/14 days with Remdesivir reduced cardiovascular events by interrupting cytokines signaling pathways (IL-2, IL-6, IL-10, and IFN-Y), resulting in a reduced immune cell function and hyperinflammation. This combination received a new Emergency Use Authorization from the FDA (Calabrese and Calabrese, 2021). In the light of these detailed evidence, the picture of JAK/STAT, especially at the cardiac level, appears to be extremely peculiar, involving different isoforms in different functions.

However, recent studies have raised concerns about the appearance of blood clots formation associated with the use of JAK inhibitors at higher doses. Therefore, the FDA has added a new boxed warning to the package insert of Tofacitinib cautioning its use in patients with cardiovascular risk treated with a dose of 10 mg twice daily (Aschenbrenner, 2019). At this dose, the Agency did not approve the drug for RA or psoriatic arthritis (PsA) but only for the treatment of the early stages of ulcerative colitis and for the long term only in limited situations.

## 7 Conclusion and future perspectives

Targeting and modulating the JAK/STAT pathway could be a valuable strategy to alleviate metabolic disease, increasing patient compliance in comparison to monoclonal antibodies or biological drugs that require subcutaneous or intravenous administration, as well as being less accessible from an economic point of view. Studies conducted so far, however, have highlighted the complexity of these molecules. While JAK inhibitors (JAKi) show promise for treating obesity and diabetes, recent reports have raised concerns about their cardiovascular safety profile. The FDA has highlighted an increased risk of serious heart-related events, cancer, blood clots and death experienced by several patients with inflammatory chronic conditions treated for long periods. However, efficacy and safety studies conducted on Baricitinib for the management of severe COVID-19 patients, including obese and with multiple comorbidities, did not reveal adverse effects and in particular thrombosis in acute use (Thoms et al., 2022). Considering the ubiquitous and pleiotropic nature of the JAK-STAT signaling pathway, directing therapy to specific organs at different stages could be beneficial (Gurzov et al., 2016). Innovations in pharmaceutical technology, may enable targeted drug delivery to specific metabolic organs, reducing the risks associated with systemic effects such as heart attacks, strokes, or serious infections. At the same time, the benefit provided at the level of districts such as the liver, skeletal muscle, adipose tissue, would benefit the individual overall, especially by improving the lipid profile and consequently the incidence of atherosclerotic events.

In conclusion, it appears clear that JAK-STAT inhibitors deserve to be considered to treat diabetic and obesity diseases, but the planning of further large-scale studies is essential in order to define their risk/benefit profile.
